# Impact of Pre-Blood Collection Factors on Plasma Metabolomic Profiles

**DOI:** 10.3390/metabo10050213

**Published:** 2020-05-21

**Authors:** Sheetal Hardikar, Richard D. Albrechtsen, David Achaintre, Tengda Lin, Svenja Pauleck, Mary Playdon, Andreana N. Holowatyj, Biljana Gigic, Petra Schrotz-King, Juergen Boehm, Nina Habermann, Stefanie Brezina, Andrea Gsur, Eline H. van Roekel, Matty P. Weijenberg, Pekka Keski-Rahkonen, Augustin Scalbert, Jennifer Ose, Cornelia M. Ulrich

**Affiliations:** 1 Population Sciences, Huntsman Cancer Institute, Salt Lake City, UT 84112, USA; rich.albrechtsen@hsc.utah.edu (R.D.A.); tengda.lin@hci.utah.edu (T.L.); svenja.pauleck@hci.utah.edu (S.P.); mary.playdon@hci.utah.edu (M.P.); andreana.holowatyj@hci.utah.edu (A.N.H.); Boehm.juergen@outlook.com (J.B.); Jennifer.ose@hci.utah.edu (J.O.); Neli.ulrich@hci.utah.edu (C.M.U.); 2 Department of Population Health Sciences, University of Utah, Salt Lake City, UT 84108, USA; 3 Cancer Prevention, Population Health Sciences, Fred Hutchinson Cancer Research Institute, Seattle, WA 19024, USA; 4 International Agency for Research on Cancer, 69372 Lyon, France; achaintred@iarc.fr (D.A.); keskip@iarc.fr (P.K.-R.); ScalbertA@iarc.fr (A.S.); 5 Department of Nutrition and Integrative Physiology, University of Utah, Salt Lake City, UT 84108, USA; 6 Department of Medicine, Vanderbilt University Medical Center, Nashville, TN 37232, USA; 7 Vanderbilt-Ingram Cancer Center, Nashville, TN 37232, USA; 8 Department of Surgery, University of Heidelberg, 69120 Heidelberg, Germany; Biljana.Gigic@med.uni-heidelberg.de; 9 Division of Preventive Oncology, National Center for Tumor Diseases (NCT) and German Cancer Research Center (DKFZ), 69120 Heidelberg, Germany; petra.schrotz-king@nct-heidelberg.de (P.S.-K.); nina.habermann@embl.de (N.H.); 10 Genome Biology, European Molecular Biology Laboratory (EMBL), 69117 Heidelberg, Germany; 11 Institute of Cancer Research, Department of Medicine I, Medical University of Vienna, 1090 Vienna, Austria; stefanie.brezina@meduniwien.ac.at (S.B.); andrea.gsur@meduniwien.ac.at (A.G.); 12 Department of Epidemiology, GROW School for Oncology and Developmental Biology, Maastricht University, 6211 LK Maastricht, The Netherlands; eline.vanroekel@maastrichtuniversity.nl (E.H.v.R.); mp.weijenberg@maastrichtuniversity.nl (M.P.W.)

**Keywords:** metabolites, metabolomics, blood collection, sample handling, processing, age, sex, smoking, alcohol, NSAID, physical activity, confounding

## Abstract

Demographic, lifestyle and biospecimen-related factors at the time of blood collection can influence metabolite levels in epidemiological studies. Identifying the major influences on metabolite concentrations is critical to designing appropriate sample collection protocols and considering covariate adjustment in metabolomics analyses. We examined the association of age, sex, and other short-term pre-blood collection factors (time of day, season, fasting duration, physical activity, NSAID use, smoking and alcohol consumption in the days prior to collection) with 133 targeted plasma metabolites (acylcarnitines, amino acids, biogenic amines, sphingolipids, glycerophospholipids, and hexoses) among 108 individuals that reported exposures within 48 h before collection. The differences in mean metabolite concentrations were assessed between groups based on pre-collection factors using two-sided *t*-tests and ANOVA with FDR correction. Percent differences in metabolite concentrations were negligible across season, time of day of collection, fasting status or lifestyle behaviors at the time of collection, including physical activity or the use of tobacco, alcohol or NSAIDs. The metabolites differed in concentration between the age and sex categories for 21.8% and 14.3% metabolites, respectively. In conclusion, extrinsic factors in the short period prior to collection were not meaningfully associated with concentrations of selected endogenous metabolites in a cross-sectional sample, though metabolite concentrations differed by age and sex. Larger studies with more coverage of the human metabolome are warranted.

## 1. Introduction

In recent years, “-omics” technologies have emerged as helpful tools for addressing population health questions within large epidemiologic data sets. Metabolomics is one such technology in which hundreds or thousands of small molecules from various biochemical pathways, and as many xenobiotics derived from exposures like diet or drugs, can be measured in biospecimens [1,2,3]. Metabolomics is particularly well-suited to the study of complex, multifactorial chronic diseases, including obesity [4], diabetes [4], Alzheimer’s disease [5] and cancer [6], and understand their etiology, build disease diagnostic models, identify biomarkers of disease prognosis, and targets for disease treatment [7,8,9,10]. As multiple large epidemiologic cohorts have measured metabolic phenotypes in their study populations, identifying the major influences on metabolite concentrations is critical to interpreting the findings from these studies [11].

An investigation of exposure phenotypes in large epidemiologic studies entails collecting biospecimens, sometimes across a large geographic region, and requires establishing standardized procedures for blood collection, taking into account participant-related factors at the time of blood collection (e.g., fasting state, smoking, alcohol use, physical activity, and medication use surrounding the collection) and extrinsic factors (e.g., time of the day, season of the year) that may directly or indirectly affect biomarker measurements [12,13,14]. These factors may vary widely between participants and across populations in epidemiologic cohorts [15], possibly contributing towards confounding effects in metabolite analysis and/or measurement errors that can distort study findings. Therefore, there is an immediate need to understand how intrinsic and extrinsic factors surrounding blood sample collection influence the metabolome.

We examined the effects of pre-blood collection factors in the short period of up to 48 hours (h) prior to blood draw on 133 targeted metabolites in blood samples from 108 individuals from the PRÄVENT cohort in Heidelberg, Germany. Our study allows simultaneous assessment of the effects of multiple pre-blood collection factors on metabolite levels in a well-annotated epidemiologic cohort. The results from this study may help inform blood collection procedures and the methodologic design of future metabolomics experiments.

## 2. Results

The characteristics of the study population are shown in Table 1. The mean age of the participants was 51.6 years (standard deviation: ±14.7) with 63.9% of the participants being female. The average age in the younger age group (≤median age) was 39.8 years, while that in the older age group (>median age) was 63.7 years. Over half (57.4%) of the participants provided a blood sample in the fall, while the remaining blood samples were evenly distributed between the remaining seasons (51.9% provided a blood sample during the high sun season, May to October). The majority (75%) of blood collections occurred in the morning and midday, and most (77.8%) fasted for less than 3 h before the blood draw, with 32.4% fasting less than 1 h prior to the blood draw. Physical activity in the 12 h prior to blood draw was reported by 18.5% of participants. A small proportion (9.3%) of participants reported using NSAIDs, while 13% reported the use of tobacco in the day prior to the blood draw. Nearly half of the participants (49.1%) consumed alcohol within 48 h prior to providing the blood sample.

**Figure 1:**
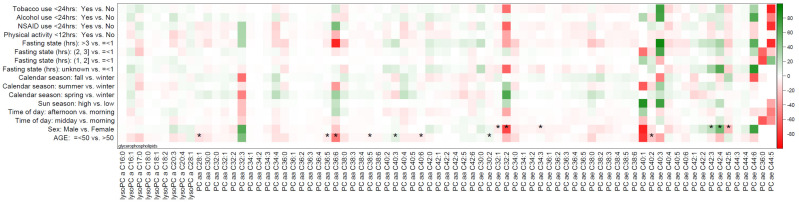
Heatmap of percentage difference in mean metabolite concentrations of glycerophospholipids across categories of pre-blood collection factors in the PRÄVENT cohort (*n* = 108). Statistically significant (adjusted for age and sex) findings based on FDR correction indicated with an “*”.

**Figure 2:**
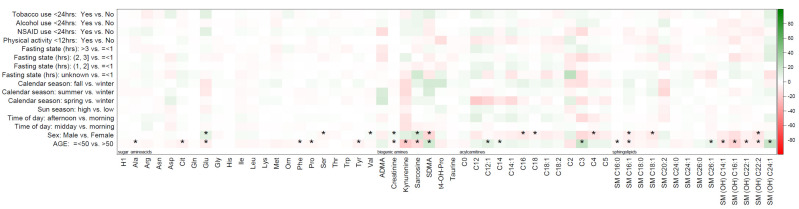
Heatmap of percentage difference in mean metabolite concentrations of acylcarnitines, amino acids and biogenic amines, sphingolipids, and hexoses across categories of pre-blood collection factors in the PRÄVENT cohort (*n* = 108). Statistically significant (adjusted for age and sex) findings based on FDR correction indicated with an “*”.

The mean (standard deviation) and median (minimum, maximum) concentrations of all the metabolites are summarized in Supplementary Materials Table S1. The percentage differences in individual metabolite concentrations between pre-blood collection factor categories against reference categories are displayed in the heat maps (Figure 1 and Figure 2). As shown in the figure, glycerophospholipids, including phosphatidylcholines alkyl-acyl (PC ae) C32:2, PC ae C36:6, PC ae C44:5, PC ae C44:6, PC ae C40:3, and PC ae C40:1, displayed the highest percentage difference in metabolite concentrations across most pre-collection factors, except for physical activity and calendar season. Table 2 summarizes the median, 10th, and 90th percentiles for mean percentage difference values in metabolite concentrations according to metabolite class for age and sex. These are aggregate changes over all the metabolites in a particular metabolite class and do not reflect changes in individual metabolite concentrations. The average of percent differences in metabolite concentrations among women were negligible for glycerophospholipids (−1.2%), amino acids and biogenic amines (0.8%), and hexoses (−1.3%) compared to men. For example, the percent difference in mean metabolite concentrations for all the metabolite concentrations averaged over all the amino acids and biogenic amines was 0.8% higher for females compared to males. The mean difference in metabolite concentrations for acylcarnitines was 6% lower (mean (10th and 90th percentile) = −6.0 (−9.6, 1.8), *p* = 0.37) among females and 6.3% higher (mean (10th and 90th percentile) = 6.3 (−1.8, 10.4), *p* = 0.42) among older adults. The percent differences between other metabolite classes by age and sex were modest, ranging from +0.8% to −3.8%, as outlined in Table 2. A summary of the percentage differences in metabolite concentrations by metabolite class for all other pre-collection factors (time of day, season, fasting state, physical activity, NSAID use, smoking, and alcohol consumption) is presented in Table 3. The percent differences in metabolite concentrations were negligible across season, time of day of collection, fasting status, or lifestyle behaviors at the time of collection, including physical activity or the use of tobacco, alcohol, or NSAIDs.

Table 4 summarizes the results of *t*-tests for differences in mean metabolite concentrations across the categories of sun season, tobacco use, alcohol intake, NSAID use, and physical activity, along with the results of ANOVA for calendar season, time of day, and fasting state (detailed in Supplementary Materials Table S2). The differences in pre-collection factors, including the time of blood collection, season, fasting state, physical activity, NSAID use, smoking, and alcohol consumption did not statistically significantly differ after adjustment for multiple comparisons. The metabolites with statistically significant differences in their concentrations by age and sex are outlined in Supplementary Materials Table S3 (age) and Supplementary Materials Table S4 (sex) and are also indicated in Figure 1 and Figure 2 with an asterisk. Briefly, the comparison of mean metabolite concentration by sex demonstrated statistically significant changes in 19 of the 133 metabolites (14.3%), while the mean metabolite comparison by age showed statistically significant changes in 29 of the 133 metabolites (21.8%). No specific pattern for statistically significantly different metabolite class by age and sex was observed, with every metabolite class having at least some metabolites that were statistically significant after a multiple testing correction. Metabolites significantly different by age and sex included creatinine and sarcosine (amino acids and biogenic amines), SM_C16:1 (sphingolipid), and phosphatidylcholine alkyl-acyl C40:3.

## 3. Discussion

In this cross-sectional study, we utilized data from a cohort of 108 individuals to investigate whether human biological, lifestyle, and biospecimen-related factors up to 48 h prior to blood collection relate to variability in plasma concentrations of 133 targeted plasma metabolites. We observed that metabolite concentrations significantly differed between men and women, and age groups (younger versus older adults), particularly for acylcarnitines and glycerophospholipids. Other short-term pre-collection factors (i.e., time of day of blood collection, season, hours of fasting, physical activity, NSAID use, tobacco use, and alcohol consumption) were not associated with significant differences in mean metabolite concentrations across variable categories.

Recent studies have investigated the value of standardizing metabolomic measurement and analysis protocols, from sample collection to assay quantification [16,17]. In epidemiological analyses, controlling for variables such as blood collection techniques, biospecimen transport method, biospecimen processing times, and storage conditions can help to preserve the stability of metabolites in a sample, along with the increasing accuracy and validity of metabolite measurements [16,17]. However, variation in participant behaviors shortly prior to the biospecimen collection may also influence metabolite measurement and, therefore, warrants consideration.

Human biologic factors, including age and sex, may affect the measurement of metabolite concentrations in plasma. Lawton et al. conducted untargeted metabolomics on plasma samples from a cohort of 269 healthy individuals to examine metabolomic variability based on participant age, sex, and race [18]. They observed that more than a third of annotated metabolites varied significantly by age; similar variation was also observed by sex, albeit for fewer metabolites. Pitkanen et al. identified age- and sex-related variation in serum amino acids in a cohort of 72 healthy participants [19]. Their study reported age-related decreases in essential, non-essential, and branched-chain amino acids, and a significant difference by sex, such that men had higher levels of amino acids than women [19]. Sampson et al. observed statistically significant correlations of age, sex, and fasting status with 385 metabolites (amino acids, carbohydrates, fatty acids, androgens, and xenobiotics) measured in repeated plasma samples among 184 men and women [20]. They concluded that a very small proportion of variability in their measured metabolite concentrations was attributable to variations in these factors, with the majority of the variability attributed to inter-person variability [20]. The differences observed in metabolite concentrations by sex could also be attributed to hormonal contraceptive use among females. A recent study by Sales et al. observed a strong impact of hormonal contraceptives on the female plasma lipidome, such that the lipidomes of women that use hormonal contraceptives were statistically significantly different from those of women not reporting hormonal use [21]. It is plausible that contraceptive use may modify the associations of metabolite concentrations by sex in the current study, however, we were unable to account for this difference in our analysis. Taken together, age and sex may be important factors contributing to the variability of metabolite concentrations (particularly of amino acids) and should be accounted for in metabolomic analyses of epidemiologic studies.

Few studies have examined the influence of multiple short-term pre-collection factors on metabolomics in a single epidemiologic cohort. A recent study by Townsend et al. quantified 166 plasma metabolites from 423 healthy participants and examined potential sources of metabolomic variability [15]. Their results are in agreement with our findings for acylcarnitines, amines, and amino acid metabolic classes, suggesting that fasting time, time of day of blood collection, and season of blood draw did not strongly influence variability in the studied metabolite levels for these classes. They reported significant differences in metabolite concentrations for organic acids, purines and pyrimidines, bile acids, and vitamins by time of the day, fasting status, and season of blood collection, however, these metabolites were not included in our targeted analysis. Using untargeted metabolomics, Kim et al. evaluated the blood plasma and urine samples of 26 volunteers for the effect of time of collection and postprandial duration [14], reporting sample preparation and analysis to be a larger source of variability than the meals and time of day. Brauer et al. examined the effects of fasting time on blood amino acids and acylcarnitines in 10 adults and found that amino acid metabolite levels may be affected by fasting for a period longer than 5 h [12]. Furthermore, while our cohort included a much larger sample size compared to Brauer et al., the participants were not directly instructed to fast, and subsequently very few participants fasted for over 5 h, making comparisons difficult. Our selected plasma metabolites do not appear to be influenced by extrinsic factors around the time of blood collection, such as time of the day or calendar season of blood collection. Additionally, for the selected plasma metabolites evaluated in this study, imposing blood collection requirements such as fasting does not seem to influence the variability in metabolite concentrations, at least for the metabolite classes we evaluated.

Most studies to date that have investigated the impact of physical activity on metabolite levels have focused on a longer exposure period of months to years in the pre-diagnostic period [22,23,24,25], or examined the immediate or real-time effects of physical activity on metabolites with unequivocal results [25]. Our study examined the effects of physical activity within a 12 h time period prior to blood collection and we did not observe any association with the metabolites studied. Previous animal studies have examined the effects of NSAIDs on metabolite levels. Montrose et al. demonstrated that NSAIDs, such as celecoxib, may lead to reductions in stool metabolite concentrations of glucose, amino acids, and lipids in mice [26]. We did not observe any significant differences by NSAID use among a subset of metabolites studied, suggesting that the intake of NSAIDs in the immediate period (12–24 h) prior to blood draw does not affect the metabolite concentrations for the metabolites we measured. Thus, the metabolomic profiles for our selected metabolites may still be valid even when epidemiologic studies did not impose any restrictions on the intake of anti-inflammatory drugs before blood draws.

Previous studies have investigated serum metabolite levels in relation to tobacco use to understand their role in disease processes [27,28]. A recent study assessed the variation in nicotine metabolism ratio (NMR, ratio of hydroxycotinine to cotinine) by genetic, hormonal, and demographic factors [29] among adults that smoked over 10 cigarettes per day. The authors reported a statistically significant association of NMR with cigarette smoking and alcohol use. However, this study assessed the influence of longer-term exposure to smoking and alcohol, but did not specifically test the effect of these exposures in the short period prior to blood collection. Alcohol-related metabolome alterations have been previously associated with oxidative stress [30,31] and diseases, including cancers [32,33]. However, we do not report any significant differences in studied metabolites by alcohol or tobacco consumption around blood sample collection. This suggests that alcohol and tobacco use in the 48 h prior to blood sample collection is not associated with variability in the studied metabolites.

An important limitation to our study is the use of targeted metabolomics, limiting our coverage of the metabolome. Our sample size was modest to evaluate pre-blood collection factor subcategories, particularly given the number of exposures evaluated. Our study cohort consisted of cancer-free individuals, however, we did not have information on other comorbidities or chronic conditions, such as diabetes, that may further influence plasma metabolomic profiles. Lastly, we were limited in assessing the effect on metabolite concentrations by the pre-blood collection factor questionnaires that were administered as part of this study. We focused on the assessment of the short-term effects of a set of robust patient, behavioral, and external factors that can be easily assessed in large-scale clinical or epidemiologic studies with a pre-blood collection questionnaire. This does not diminish the possibility that such factors may affect metabolite profiles in the long term. We recognize that there may be other important sources of metabolite variability that were not accounted for in our pre-blood collection questionnaire, such as dietary factors or the intake of vitamin supplements. Our study has several strengths, notably having data derived from a well-annotated cohort of cancer-free individuals with information collected within a specific window prior to blood draw, as well as precise standardized sampling and analysis protocols for metabolite quantification. Our study is also unique in its consideration of a large number of pre-collection factors within a single cohort, compared with previous studies that evaluated one or two pre-collection factors.

In conclusion, we found that age and sex, factors usually available at hand for epidemiologic cohorts, are important intrinsic adjustment factors to account for variations in concentrations of the studied metabolites. Researchers could address the influence of age and sex in a number of ways: 1) appropriate age and sex matching; 2) statistical adjustment for these variables; or 3) investigations within a narrow age window. Other extrinsic blood collection factors and lifestyle behaviors in the short period of 48 h immediately prior to collection do not appear to contribute to significant variability in the selected, frequently-measured plasma metabolite concentrations. Studies in larger cohorts with greater coverage of the human metabolome are needed to ensure that epidemiologic cohorts with archived blood samples may produce robust metabolomics data on metabolites beyond those measured here, even if strict blood collection protocols or detailed lifestyle exposure information at the time of blood collection are not readily available.

## 4. Materials and Methods

Study Population: The current analysis was performed with data collected from *n* = 108 cancer-free control participants recruited in Heidelberg, Germany as part of the PRÄVENT cohort, a control cohort of the ColoCare Study, a multicenter international prospective cohort recruiting newly diagnosed stage I–IV colorectal cancer patients with the aim of investigating the predictors of cancer recurrence, treatment toxicities, survival, and health-related quality of life (ClinicalTrials.gov: NCT02328677) [34]. Study approval for the PRÄVENT cohort has been obtained from the Institutional Review Board of the Medical Faculty at the University of Heidelberg. Written informed consent was obtained from all study participants. Information on demographic and lifestyle factors was collected via a comprehensive questionnaire; anthropometric measurements were collected on all study participants. Venous blood samples (45 mL) were collected from each participant recording the date and time of sample collection. Additionally, all study participants completed a questionnaire reporting on pre-collection exposures at the time of blood draw.

Blood Collection Questionnaire: Study participants completed a short questionnaire that collected information on: the number of hours since food consumption, participation in physical activity in the last 12 h, any non-steroidal anti-inflammatory drugs (NSAID) use in the last 24 h, any tobacco use in the last 24 h, and alcohol consumption in the last 48 h. Participants reported whether exposures occurred within the specified timeframe (“yes” or “no”) and were provided the opportunity to report specific activities performed or substances consumed. ColoCare Study staff contacted PRÄVENT participants by phone to fill in any missing data wherever possible.

Biospecimen Handling, Data Acquisition, and Data Preprocessing: Blood samples were collected in EDTA tubes and processed within 4 h of collection and stored at −80 °C. Plasma samples were shipped on dry ice to the International Agency for Research on Cancer (IARC) in Lyon, France for metabolite analyses. Details on the sample analysis have been previously described [35,36,37,38]. The samples were analyzed using a targeted metabolomics kit (AbsoluteIDQ™ p180 kit: BIOCRATES Life Sciences AG, Innsbruck, Austria) designed for the analysis of 186 metabolites from five compound classes. Amino acids and biogenic amines were quantified by ultrahigh performance liquid chromatography coupled to tandem mass spectrometry (UHPLC-MS/MS), whereas lipids, sugar, and acylcarnitines were semi-quantified by flow injection analysis on the same mass spectrometer (FIA-MS/MS). Samples were prepared using the materials and methods provided with the kit, resulting in two separate samples for the LC-MS/MS analysis (phenylthiocarbamyl-derivatized amino acids and biogenic amines) and FIA-MS/MS analysis (lipids, acylcarnitines, and hexoses). For the LC-MS/MS analysis, 5 µL of the sample was injected on an ACQUITY UPLC BEH C18 column (2.1 × 75 mm, 1.7 µm) with a BEH C18 VanGuard pre-column (2.1 × 5 mm, 1.7 µm) (Waters, Milford, MA, USA). The mobile phase consisted of ultrapure water (A) and acetonitrile (B), both with 0.2% formic acid. The gradient profile was as follows: 0–0.38 min: 0% B; 0.38–3 min: 0 > 15% B; 3–5.4 min: 15 > 70% B; 5.4–5.5 min: 70 > 100% B; 5.5–5.93 min: 100% B; 5.93–6.1 min: 100 > 0% B; 6.1–6.6 min: 0% B. The flow rate was 0.9 mL/min (0.99 mL/min between 5.66 and 5.84 min). The column temperature was 50 °C. FIA-MS/MS analysis was achieved by isocratic elution without a chromatographic column using a kit-provided FIA running solvent.

Statistical Analysis: Metabolites with a coefficient of variation above 20% on quality control samples, and with > 20% missing values (defined as values less than the limit of detection (LOD) or lower limit of quantification (LLOQ), or truly missing due to failed analysis) in study samples were excluded from further analysis. After exclusions, a total of 133 metabolites out of the 186 measured (71.5%) were retained for further statistical analysis. The metabolite concentrations were log2 transformed to achieve a normal distribution. Metabolites were classified by their structure and biologic function into acylcarnitines (n = 13), amino acids and biogenic amines (n = 28), sphingolipids (n = 14), glycerophospholipids (n = 77), and hexose (n = 1). Data on pre-collection factors were categorized into relevant groups (age: above vs. below (reference) median, sex: male vs. female (reference), time of blood draw: morning (7–10 am) (reference) vs. midday (10 am–2 pm), vs. afternoon (2–4 pm), season: winter (Dec.–Feb.) (reference) vs. spring (Mar.–May) vs. summer (Jun.–Aug.) vs. fall (Sep.–Nov.) as well as low sun (Nov.–Apr.) (reference) vs. high sun (May–Oct.), physical activity: yes vs. no (reference), NSAID use: yes vs. no (reference), tobacco use: yes vs. no (reference), alcohol consumption: yes vs. no (reference), and fasting status in hours: <1, 1 to < 2, 2 to < 3 vs. 3 (reference)).

For each pre-blood collection factor, we calculated the mean percentage difference in concentration of 133 metabolites by comparing the metabolite concentration within each pre-blood collection factor category to the referent category for that factor, as described above. For example, for NSAID use, we calculated the difference in mean metabolite concentration for each metabolite among those reporting NSAID use as compared to non-users, and then computed this difference as a percentage. A heat map displaying the mean percentage difference in metabolite concentrations across pre-collection factors was created. Additionally, we summarized the percentage differences within each metabolite category (i.e., acylcarnitines, amino acids and biogenic amines, sphingolipids, glycerophospholipids, and hexoses). The differences in mean metabolite concentrations were evaluated by two-sided *t*-tests (dichotomous variables), and analysis of variance tests or ANOVA (> 2 groups); both unadjusted and age- and sex-adjusted differences (for factors other than age/sex) in mean metabolite concentrations were computed. Raw and adjusted *p*-values for all analyses were ranked and corrected for multiple testing using a False Discovery Rate (FDR) correction [39]. All data analyses were conducted with SAS Studio software (SAS Studio v3.8, Cary, NC, USA).

## Figures and Tables

**Table 1:** Characteristics and pre-blood collection exposures for participants from the PRÄVENT cohort (*n* = 108).

Characteristics	Participants (*n* = 108)
Age, mean ± SD	51.6 ± 14.7
Sex	
Female, *n* (%)	69 (63.9)
Male, *n* (%)	39 (36.1)
BMI kg/m^2^ (mean ± SD)	24.4 ± 4.8
**Pre-blood collection exposures (yes/no), *n* (%)**	
Physical Activity in the last 12 h	20 (18.5)
NSAID use in the last 24 h	10 (9.3)
Tobacco use in the last 24 h	14 (13.0)
Alcohol use in the last 48 h	53 (49.1)
**Fasting state at blood draw (h), *n* (%) ^a^**	
<1	35 (32.4)
≥ 1 to < 2	33 (30.6)
≥ 2 to < 3	16 (14.8)
3 or more	20 (18.5)
**Date of blood draw, *n* (%)**	
Season	
Spring (March–May)	16 (14.8)
Summer (June–August)	13 (12.0)
Fall (September–November)	62 (57.4)
Winter (December–February)	17 (15.7)
Sun Exposure	
High Sun (May–October)	56 (51.9)
Low Sun (November–April)	52 (48.1)
**Time of blood draw, *n* (%)**	
Morning (7–10 am)	40 (37.0)
Mid-Day (10 am–1 pm)	41 (38.0)
Afternoon (1–4 pm)	27 (25.0)

^a^ There were four study participants for whom the duration of fasting was unknown.

**Table 2:** Median (10th percentile, 90th percentile) percentage differences in mean metabolite concentrations by age and sex within metabolite class in the PRÄVENT cohort (*n* = 108).

		Metabolite Categories				
		Acylcarnitines	AA and Biogenic Amines	Sphingolipids	Glycerophospholipids	Hexoses
		(*n* = 13)	(*n* = 28)	(*n* = 14)	(*n* = 77)	(*n* = 1)
Characteristic	*n* (%)	% difference in median (10th, 90th percentile)				% difference
**Sex**						
Male	39 (36.1%)	Ref	Ref	Ref	Ref	Ref
Female	69 (63.9%)	−6.0 (−9.6, 1.8)	0.8 (−2.7, 6.7)	−3.2 (−9.7, 4.1)	−1.2 (−12.0, 12.7)	−1.3
**Age**						
Below median	55 (50.9%)	Ref	Ref	Ref	Ref	Ref
Above median	53 (49.1%)	6.3 (−1.8, 10.4)	−2.7 (−6.1, −0.1)	−3.8 (−8.5, 7.2)	−2.7 (−16.9, 4.9)	−1.3

AA: Amino acids.

**Table 3:** Age- and sex-adjusted median (10th percentile, 90th percentile) percentage differences in mean metabolite concentrations by pre-blood collection factors within metabolite class in the PRÄVENT cohort (*n* = 108).

		Metabolite Categories				
		Acylcarnitines	AA and Biogenic Amines	Sphingolipids	Glycerophospholipids	Hexoses
		(*n* = 13)	(*n* = 28)	(*n* = 14)	(*n* = 77)	(*n* = 1)
Pre-blood Collection Factors	*n* (%)	% difference median (10th, 90th percentile)				% difference
**Season of Blood Collection**						
Low Sun Months (November–April)	52 (48.1%)	Ref	Ref	Ref	Ref	Ref
High-Sun Months (May–October)	56 (51.9%)	2.7 (0.6, 5.7)	0.3, (−2.4, 2.0)	0.4 (−2.7, 2.2)	0.5 (−2.1, 8.0)	−1.1
Winter (December–February)	17 (15.7%)	Ref	Ref	Ref	Ref	Ref
Spring (March–May)	16 (14.8%)	2.1 (−9.8, 7.3)	1.6 (−1.8, 2.0)	1.2 (−4.6, 5.0)	1.7 (−9.9, 11.7)	−1.9
Summer (June–August)	13 (12.0%)	−4.7 (−15.8, 3.6)	0.2 (−1.9, 2.0)	1.9 (−0.1, 2.7)	2.9 (−6.2, 16.9)	−0.1
Fall (September–November)	62 (57.4%)	1.8 (−2.8, 3.2)	−0.3 (−2.3, 2.0)	1.1 (−3.4, 6.1)	0.4 (−9.1, 7.6)	−2.1
**Time of Day of Blood Collection**						
Morning (7–10 am)	40 (37.0%)	Ref	Ref	Ref	Ref	Ref
Midday (10 am–1 pm)	41 (38.0%)	0.6 (−2.0, 2.6)	−0.7 (−3.6, 1.1)	−0.2 (−1.7, 1.4)	−1.3 (−5.4, 2.2)	−0.2
Afternoon (1–4 pm)	27 (25.0%)	3.5 (−6.1, 8.3)	1.1 (−2.1, 3.0)	−0.4 (−2.4, 2.5)	−1.2 (−8.7, 3.3)	0.5
**Fasting at Blood Collection (h) ^a^**						
3 or more	20 (18.5%)	Ref	Ref	Ref	Ref	Ref
≥ 2 to < 3	16 (14.8%)	2.6 (−3.3, 8.1)	−1.0 (−3.9, 2.0)	−0.8 (−4.1, 3.4)	−2.7 (−19.2, 16.4)	0.3
≥ 1 to < 2	33 (30.6%)	1.5 (−8.2, 4.1)	−0.5 (−4.4, 2.0)	−1.5 (−5.7, 6.1)	0.6 (−7.5, 5.9)	0.0
<1	35 (32.4%)	1.2 (−1.8, 7.2)	−0.2 (−2.3, 2.0)	0.6 (−2.5, 3.2)	0.5 (−2.7, 3.8)	0.8
**Modifiable exposures at Blood Collection**						
No NSAID Use (<24 h)	98 (90.7%)	Ref	Ref	Ref	Ref	Ref
NSAID Use (<24 h)	10 (9.3%)	1.7 (−5.9, 7.5)	1.7 (−1.0, 6.1)	−3.0 (−5.0, −0.4)	−1.6 (−10.1,11.2)	2.0
No Tobacco Use (<24 h)	94 (87.0%)	Ref	Ref	Ref	Ref	Ref
Tobacco Use (<24 h)	14 (13.0%)	2.5 (−0.9, 5.7)	−0.1 (−2.5, 7.6)	−0.7 (−3.1, 2.8)	−0.5 (−7.0, 6.7)	−0.2
No Physical Activity (<12 h)	88 (81.5%)	Ref	Ref	Ref	Ref	Ref
Physical Activity (<12 h)	20 (18.5%)	−3.2 (−7.6, 2.3)	−0.1 (−2.6, 1.5)	−1.9 (−5.9, 4.0)	−2.7 (−10.7, 1.3)	−0.4
No Alcohol Use (<48 h)	55 (50.9%)	Ref	Ref	Ref	Ref	Ref
Alcohol Use (<48 h)	53 (49.1%)	−0.1 (−2.1, 5.4)	−0.7 (−2.2, 1.8)	−0.2 (−4.1, 5.3)	1.4 (−4.9, 12.5)	−0.4

AA: Amino acids; ^a^ There were four study participants with an unknown fasting duration prior to blood collection.

**Table 4:** Total number of metabolites within metabolite classes demonstrating statistically significant metabolite concentrations according to pre-blood collection factors in the PRÄVENT cohort (*n* = 108) ^a^.

Comparison Groups	Acylcarnitines	AA and Amines	Sphingolipids	Glycerophospholipids	Hexoses
	(*n* = 13)	(*n* = 28)	(*n* = 14)	(*n* = 77)	(*n* = 1)
Sex (male vs female), *n* (%)	3 (23.1%)	6 (21.4%)	3 (21.4%)	7 (9.1%)	0
Age (above vs below median), *n* (%)	3 (23.1%)	10 (35.7%)	8 (57.1%)	8 (10.39%)	0
Pre-Collection Factors ^b^	0	0	0	0	0

AA: Amino acids; ^a^ All comparisons were adjusted for multiple testing using the FDR correction; ^b^ Differences in pre-collection factors, including time of blood collection, season, fasting state, physical activity, NSAID use, smoking, and alcohol consumption did not statistically significantly differ for any of the metabolites within any metabolite class after adjustment for multiple comparisons.

## Supplementary Materials

The following are available online at https://www.mdpi.com/2218-1989/10/5/213/s1, Table S1: Mean (standard deviation) and median (minimum, maximum) concentrations of all the metabolites across all samples, Table S2: Age- and sex-adjusted *p*-values for mean metabolite levels for all 133 metabolites for pre-collection factors (excluding age and sex) in the PRÄVENT cohort (*n* = 108), Table S3: Metabolites with significantly different mean concentrations by age categories in the PRÄVENT cohort (*n* = 108), Table S4: Metabolites with significant differences in mean metabolite concentrations by sex categories in the PRÄVENT cohort (*n* = 108).
