# Supplementary material for: Impact of Pre-Blood Collection Factors on Plasma Metabolomic Profiles
Source: Metabolites. 2020 May 21;10(5):213. doi: 10.3390/metabo10050213 (PMC7281389; doi:10.3390/metabo10050213)
Supplement: Supplementary file 1 [file metabolites-10-00213-s001.pdf]

Supplementary tables for

Impact of pre-blood collection factors on plasma metabolomic profiles

Sheetal Hardikar[1,2,3*], Richard D. Albrechtsen[1], David Achaintre[4], Tengda Lin[1,2], Svenja Pauleck[1], Mary Playdon[1,5], Andreana N. Holowatyj[1,2,6,7], Biljana Gigic[8], Petra Schrotz-King[9], Juergen Boehm[1], Stefanie Brezina[10], Andrea Gsur[10], Eline H. van Roekel[11], Matty P. Weijenberg[11], Pekka Keski-Rahkonen[4], Augustin Scalbert[4], Jennifer Ose[1,2&], Cornelia M. Ulrich[1,2&]

[1]Population Sciences, Huntsman Cancer Institute, Salt Lake City, Utah

[2]Department of Population Health Sciences, University of Utah, Salt Lake City, Utah

[3]Cancer Prevention, Population Health Sciences, Fred Hutchinson Cancer Research Institute, Seattle, Washington

[4]International Agency for Research on Cancer, Lyon, France

[5]Department of Nutrition and Integrative Physiology, University of Utah, Salt Lake City, Utah

[6]Department of Medicine, Vanderbilt University Medical Center, Nashville, Tennessee

[7]Vanderbilt-Ingram Cancer Center, Nashville, Tennessee

[8]Department of Surgery, University of Heidelberg, Germany

[9]Division of Preventive Oncology, National Center for Tumor Diseases (NCT) and German Cancer Research Center (DKFZ), Heidelberg, Germany

[10]Institute of Cancer Research, Department of Medicine, Medical University of Vienna, Austria.

[11]Department of Epidemiology, GROW School for Oncology and Developmental Biology, Maastricht University, Maastricht, The Netherlands.

* Correspondence: sheetal.hardikar@hci.utah.edu; 801-213-6238

[&] Authors contributed equally

Table S1. Mean (standard deviation) and median (minimum, maximum) concentrations of all the metabolites across all samples

Class	Metabolite	Samples (n)	Mean ± Standard Deviation	Median (Min, Max)
Acylcarnitines	C0	108	34.1 ± 7.53	34.1 (16.9, 54.1)
	C12	71	0.1 ± 0.05	0.1 (0.1, 0.3)
	C12:1	79	0.2 ± 0.05	0.1 (0.1, 0.4)
	C14	69	0.0 ± 0.01	0.0 (0.0, 0.1)
	C14:1	108	0.1 ± 0.04	0.1 (0.0, 0.2)
	C16	108	0.1 ± 0.03	0.1 (0.1, 0.3)
	C18	105	0.0 ± 0.01	0.0 (0.0, 0.1)
	C18:1	108	0.1 ± 0.05	0.1 (0.0, 0.4)
	C18:2	107	0.0 ± 0.02	0.0 (0.0, 0.1)
	C2	108	5.8 ± 2.32	5.3 (2.3, 12.1)
	C3	108	0.4 ± 0.13	0.4 (0.2, 0.8)
	C4	108	0.2 ± 0.07	0.2 (0.1, 0.4)
	C5	99	0.2 ± 0.05	0.1 (0.1, 0.3)
Amino Acids and Biogenic Amines	ADMA	108	0.4 ± 0.10	0.4 (0.3, 0.9)
	Alanine	108	386.2 ± 114.72	373.5 (139.0, 851.0)
	Arginine	108	71.0 ± 23.38	69.3 (9.9, 140.0)
	Asparagine	108	48.4 ± 11.38	47.1 (31.7, 108.0)
	Aspartic acid	76	7.3 ± 2.02	7.0 (5.0, 15.7)
	Citrulline	108	31.9 ± 8.18	31.7 (10.3, 53.6)
	Creatinine	108	72.5 ± 13.16	70.8 (45.7, 109.0)
	Glutamine	108	701.9 ± 109.56	713.5 (365.0, 970.0)
	Glutamate	108	38.8 ± 20.27	34.4 (13.2, 114.0)
	Glycine	108	222.1 ± 70.51	211.0 (113.0, 456.0)
	Histidine	108	78.8 ± 11.95	77.9 (46.2, 112.0)
	Isoleucine	108	72.2 ± 25.60	65.6 (29.2, 169.0)
	Kynurenine	108	2.5 ± 0.74	2.5 (1.4, 4.5)
	Leucine	108	132.5 ± 45.06	122.0 (62.4, 289.0)
	Lysine	108	201.6 ± 53.88	193.5 (106.0, 461.0)
	Methionine	108	21.5 ± 7.67	20.0 (11.3, 64.7)
	Ornithine	108	79.9 ± 30.64	72.5 (26.5, 270.0)
	Phenylalanine	108	61.5 ± 13.93	59.8 (39.3, 118.0)
	Proline	108	201.9 ± 70.22	187.5 (71.3, 403.0)
	Sarcosine	108	2.5 ± 0.74	2.4 (1.4, 7.0)
	SDMA	108	0.5 ± 0.09	0.4 (0.2, 0.6)
	Serine	108	100.9 ± 25.47	97.6 (49.7, 201.0)
	T4_OH_Pro	108	7.5 ± 3.41	6.9 (2.9, 18.4)
	Taurine	108	46.8 ± 15.79	44.0 (24.3, 144.0)
	Threonine	108	118.3 ± 30.26	117.5 (56.9, 275.0)

	Tryptophan	108	62.5 ± 15.16	60.1 (21.5, 111.0)
	Tyrosine	108	74.2 ± 26.80	68.7 (31.2, 170.0)
	Valine	108	241.0 ± 67.67	231.0 (147.0, 520.0)
Hexose	H1	108	4600.1 ± 1285.66	4493.5 (1642.0, 13715.0)
Sphingolipids	SM__OH__C14_1	108	5.1 ± 1.32	4.9 (2.5, 10.0)
	SM__OH__C16_1	108	2.2 ± 0.55	2.2 (1.3, 3.7)
	SM__OH__C22_1	108	5.7 ± 1.33	5.5 (3.0, 10.1)
	SM__OH__C22_2	108	5.0 ± 1.18	4.8 (3.1, 8.3)
	SM__OH__C24_1	108	0.4 ± 0.11	0.4 (0.2, 0.7)
	SM_C16:0	108	74.6 ± 12.87	73.6 (50.1, 119.0)
	SM_C16:1	108	10.5 ± 2.12	10.3 (6.5, 15.0)
	SM_C18:0	108	14.2 ± 3.07	13.7 (8.4, 23.8)
	SM_C18:1	108	6.7 ± 1.66	6.6 (3.7, 11.5)
	SM_C20:2	102	0.1 ± 0.06	0.1 (0.0, 0.3)
	SM_C24:0	108	7.5 ± 1.50	7.6 (3.8, 12.0)
	SM_C24:1	108	15.0 ± 3.47	14.8 (7.6, 26.4)
	SM_C26:0	108	0.0 ± 0.01	0.0 (0.0, 0.1)
	SM_C26:1	108	0.1 ± 0.03	0.1 (0.1, 0.2)
Glycerophospholipids	LysoPC_a_C16:0	108	98.3 ± 23.80	95.0 (59.6, 193.0)
	LysoPC_a_C16:1	108	2.9 ± 0.92	2.7 (1.2, 6.1)
	LysoPC_a_C17:0	108	2.0 ± 0.72	1.9 (0.8, 6.2)
	LysoPC_a_C18:0	108	28.5 ± 8.00	28.8 (13.9, 67.1)
	LysoPC_a_C18:1	108	22.0 ± 6.52	21.1 (8.3, 39.9)
	LysoPC_a_C18:2	108	33.5 ± 12.70	31.6 (6.7, 75.1)
	LysoPC_a_C20:3	108	2.2 ± 0.71	2.1 (0.7, 3.8)
	LysoPC_a_C20:4	108	5.7 ± 1.92	5.2 (2.9, 13.8)
	LysoPC_a_C28:1	108	0.3 ± 0.07	0.3 (0.1, 0.5)
	PC_ aa _C28:1	108	3.3 ± 0.85	3.3 (1.7, 6.5)
	PC_ aa _C30:0	108	5.0 ± 1.78	4.9 (1.7, 11.0)
	PC_ aa _C32:0	108	24.5 ± 5.52	23.7 (14.4, 39.7)
	PC_ aa _C32:1	108	33.5 ± 19.65	28.3 (10.1, 117.0)
	PC_ aa _C32:2	108	8.6 ± 3.29	8.3 (1.7, 23.8)
	PC_ aa _C32:3	108	0.9 ± 0.23	0.8 (0.4, 1.5)
	PC_ aa _C34:1	108	353.2 ± 85.98	343.5 (183.0, 607.0)
	PC_ aa _C34:2	108	606.9 ± 90.77	611.5 (348.0, 844.0)
	PC_ aa _C34:3	108	27.2 ± 8.89	25.2 (12.1, 58.8)

PC_ aa _C34:4	108	3.3 ± 1.48	3.0 (1.0, 7.8)
PC_ aa _C36:0	108	3.1 ± 0.86	3.0 (1.7, 5.5)
PC_ aa _C36:1	108	65.0 ± 20.13	64.0 (29.2, 151.0)
PC_ aa _C36:2	108	338.1 ± 62.95	338.5 (180.0, 473.0)
PC_ aa _C36:3	108	192.8 ± 45.76	187.5 (74.6, 351.0)
PC_ aa _C36:4	108	263.4 ± 71.22	268.0 (147.0, 463.0)
PC_ aa _C36:5	108	39.1 ± 19.92	35.1 (9.8, 120.0)
PC_ aa _C36:6	108	1.5 ± 0.57	1.4 (0.4, 3.1)
PC_ aa _C38:0	108	2.6 ± 0.80	2.5 (1.4, 5.2)
PC_ aa _C38:3	108	53.2 ± 15.60	50.1 (24.8, 104.0)
PC_ aa _C38:4	108	120.5 ± 35.27	121.5 (62.6, 244.0)
PC_ aa _C38:5	108	60.7 ± 16.74	61.1 (31.0, 99.8)
PC_ aa _C38:6	108	84.2 ± 27.22	81.5 (35.5, 170.0)
PC_ aa _C40:2	108	0.2 ± 0.07	0.2 (0.1, 0.5)
PC_ aa _C40:3	108	0.4 ± 0.09	0.4 (0.2, 0.7)
PC_ aa _C40:4	108	3.1 ± 0.98	2.8 (1.5, 7.7)
PC_ aa _C40:5	108	7.7 ± 2.50	7.5 (3.2, 15.8)
PC_ aa _C40:6	108	23.1 ± 8.04	21.8 (8.3, 48.0)
PC_ aa _C42:0	108	0.3 ± 0.09	0.3 (0.2, 0.5)
PC_ aa _C42:1	108	0.2 ± 0.04	0.2 (0.1, 0.3)
PC_ aa _C42:2	108	0.1 ± 0.03	0.1 (0.1, 0.2)
PC_ aa _C42:4	108	0.1 ± 0.02	0.1 (0.1, 0.2)
PC_ aa _C42:5	108	0.2 ± 0.07	0.2 (0.1, 0.5)
PC_ aa_ C42:6	106	0.4 ± 0.09	0.4 (0.2, 0.7)
PC_ ae _C30:0	108	0.4 ± 0.12	0.4 (0.2, 0.7)
PC_ ae _C30:2	108	0.1 ± 0.02	0.1 (0.0, 0.1)
PC_ ae _C32:1	108	5.0 ± 1.11	4.9 (2.6, 7.9)
PC_ ae _C32:2	108	1.2 ± 0.29	1.2 (0.8, 2.4)
PC_ ae _C34:0	108	2.5 ± 0.74	2.4 (1.0, 5.0)
PC_ ae _C34:1	108	18.5 ± 4.11	17.7 (10.8, 28.1)
PC_ ae _C34:2	108	20.8 ± 5.27	19.6 (8.7, 34.2)
PC_ ae _C34:3	108	13.8 ± 3.78	13.3 (4.5, 24.0)
PC_ ae _C36:0	108	1.0 ± 0.31	1.0 (0.6, 2.2)
PC_ ae _C36:1	108	12.6 ± 2.99	12.2 (6.4, 20.3)
PC_ ae _C36:2	108	22.9 ± 5.59	22.9 (10.2, 39.1)
PC_ ae _C36:3	108	12.0 ± 3.03	11.5 (4.0, 20.2)

PC_ ae _C36:4	108	24.2 ± 7.30	23.4 (12.0, 54.5)
PC_ ae _C36:5	108	16.0 ± 4.59	15.8 (8.0, 29.9)
PC_ ae _C38:0	108	2.3 ± 0.72	2.3 (1.2, 4.9)
PC_ ae _C38:2	108	2.3 ± 0.54	2.3 (0.9, 4.0)
PC_ ae _C38:3	108	5.1 ± 1.23	5.0 (2.4, 8.3)
PC_ ae _C38:4	108	16.3 ± 3.74	15.8 (9.8, 26.8)
PC_ ae _C38:5	108	20.8 ± 4.88	20.4 (11.2, 34.3)
PC_ ae _C38:6	108	8.6 ± 2.36	8.5 (4.5, 14.1)
PC_ ae _C40:1	108	1.1 ± 0.27	1.1 (0.6, 1.8)
PC_ ae _C40:2	108	1.7 ± 0.40	1.6 (1.0, 3.1)
PC_ ae _C40:3	108	0.9 ± 0.20	0.9 (0.5, 1.5)
PC_ ae _C40:4	108	2.2 ± 0.49	2.1 (1.2, 3.5)
PC_ ae _C40:5	108	3.5 ± 0.71	3.5 (2.1, 5.4)
PC_ ae _C40:6	108	4.7 ± 1.21	4.7 (2.5, 7.9)
PC_ ae _C42:1	108	0.3 ± 0.06	0.3 (0.2, 0.6)
PC_ ae _C42:2	108	0.5 ± 0.11	0.5 (0.2, 0.8)
PC_ ae _C42:3	108	0.6 ± 0.13	0.5 (0.3, 0.9)
PC_ ae _C42:4	108	0.7 ± 0.20	0.7 (0.3, 1.5)
PC_ ae _C42:5	108	1.6 ± 0.39	1.6 (0.9, 3.1)
PC_ ae _C44:3	107	0.1 ± 0.01	0.1 (0.0, 0.1)
PC_ ae _C44:4	108	0.2 ± 0.07	0.2 (0.1, 0.4)
PC_ ae _C44:5	108	1.1 ± 0.34	1.1 (0.5, 2.2)
PC_ ae _C44:6	108	0.8 ± 0.23	0.8 (0.3, 1.4)

C0 Carnitine; C12 Decanoylcarnitine; C12:1 Decenoylcarnitine; C14 Tetradecanoylcarnitine; C14:1 Tetradecenoylcarnitine; C16 Hexadecanoylcarnitine; C18 Octadecanoylcarnitine; C18:1 Octadecenoylcarnitine; C18:2 Octadecadienylcarnitine; C2 Acetylcarnitine; C3 Propionylcarnitine; C4 Butyrylcarnitine; C5 Valerylcarnitine

ADMA Asymmetric dimethylarginine

SDMA Symmetric dimethylarginine

T4_OH_Pro Trans-4-Hydroxyproline

LysoPC_a Lyso-phosphatidylcholines

PC_aa Phosphatidylcholines di-alkyl

PC_ae Phosphatidylcholines alkyl-acyl

Table S2. Age- and sex-adjusted P-values for mean metabolite levels for all 133 metabolites for pre-collection factors (excluding age and sex) in the PRÄVENT cohort (N=108).

Class	Metabolite	Time of Day (Ref: Morning)		Sun Season (Ref: Low Sun)	Calendar Season (Ref: Winter)			Fasting State (Ref: ≥3)			Physical Activity <12 Hrs (Ref: No)	NSAID Use <24 Hrs (Ref: No)	Alcohol Use <24 Hrs (Ref: No)	Tobacco Use <24 Hrs (Ref: No)
		Midday	Afternoon	High Sun	Spring	Summer	Fall	<1 Hours	1 - <2 Hours	2 - <3 Hours	Yes	Yes	Yes	Yes
Acylcarnitines	C0	0.87	0.05	0.30	0.01	0.07	0.04	0.85	0.39	0.31	0.29	0.52	0.89	0.93
	C12	0.44	0.01	0.16	0.57	0.00	0.11	0.03	0.55	0.02	0.06	0.05	0.73	0.10
	C12:1	0.43	0.69	0.12	0.46	0.00	0.04	0.01	0.62	0.09	0.18	0.01	0.47	0.23
	C14	0.87	0.06	0.83	0.69	0.00	0.49	0.16	0.97	0.14	0.21	0.55	0.37	0.21
	C14:1	0.90	0.03	0.29	0.59	0.01	0.43	0.04	0.28	0.78	0.10	0.05	0.27	0.33
	C16	0.51	0.08	0.42	0.22	0.21	0.77	0.09	0.36	0.86	0.31	0.10	0.33	0.61
	C18	0.70	0.67	0.07	0.28	0.67	0.67	0.45	0.94	0.48	0.70	0.13	0.28	0.52
	C18:1	0.79	0.06	0.63	0.42	0.35	0.69	0.09	0.23	0.52	0.20	0.20	0.69	0.87
	C18:2	0.31	0.11	0.48	0.60	0.78	0.98	0.13	0.34	0.30	0.01	0.67	0.29	0.19
	C2	0.88	0.57	0.12	0.36	0.12	0.84	0.06	0.11	0.52	0.05	0.07	0.39	0.53
	C3	0.86	0.09	0.78	0.07	0.28	0.41	0.94	0.25	0.26	0.93	0.58	0.19	0.53
	C4	0.64	0.25	0.97	0.23	0.43	0.99	0.79	0.23	0.54	0.59	0.95	0.05	0.66
	C5	0.40	0.47	0.44	0.14	0.13	0.77	0.63	0.56	0.41	0.68	0.83	0.96	0.51
Amino Acids and Biogenic Amines	ADMA	0.58	0.53	0.93	0.68	0.06	0.14	0.71	0.16	0.23	1.00	0.20	0.41	0.78
	Alanine	0.99	0.26	0.82	0.22	0.34	0.45	0.17	0.77	0.04	0.02	0.03	0.89	0.44
	Arginine	0.02	0.08	0.59	0.37	0.75	0.52	0.28	0.19	0.30	0.58	0.08	0.29	0.04
	Asparagine	0.88	0.02	0.27	0.45	0.42	0.85	0.55	0.78	0.09	0.95	0.49	0.46	0.09
	Aspartic acid	0.14	0.40	0.28	0.60	0.07	0.13	0.69	0.23	0.98	0.97	0.56	0.71	0.06
	Citrulline	0.43	0.54	0.58	0.04	0.77	0.30	0.81	0.02	0.67	0.09	0.23	0.21	0.46
	Creatinine	0.49	0.80	0.49	0.83	0.32	0.44	0.86	0.79	0.98	0.84	0.29	0.37	0.51
	Glutamine	0.60	0.40	0.16	0.07	0.98	0.45	0.59	0.85	0.71	0.28	0.68	0.54	0.72
	Glutamate	0.44	0.72	0.18	0.17	0.77	0.30	0.79	0.57	0.39	0.74	0.06	0.40	0.00
	Glycine	0.41	0.91	0.10	0.62	0.77	0.96	0.67	0.11	0.44	0.41	0.53	0.61	0.74
	Histidine	0.68	0.35	0.83	0.17	0.46	0.33	0.47	0.84	0.21	0.83	0.59	0.46	0.94
	Isoleucine	0.15	0.08	0.41	0.91	0.50	0.66	0.50	0.42	0.83	0.89	0.51	0.15	0.47
	Kynurenine	0.11	0.14	0.53	0.40	0.33	0.35	0.52	0.62	0.81	0.94	0.86	0.44	0.55
	Leucine	0.11	0.35	0.36	0.81	0.87	0.98	0.14	0.22	0.49	0.87	0.83	0.25	0.72
	Lysine	0.48	0.76	0.85	0.02	0.10	0.04	0.19	0.71	0.55	0.43	0.84	0.58	0.87
	Methionine	0.75	0.65	0.69	0.08	0.53	0.71	0.38	0.24	0.07	0.56	0.40	0.07	0.40
	Ornithine	0.57	0.80	0.41	0.46	0.91	0.68	0.79	0.43	0.03	0.83	0.84	0.56	0.01
	Phenylalanine	0.27	0.07	0.83	0.46	0.47	0.42	0.91	0.69	0.12	0.91	0.79	0.33	0.26
	Proline	0.69	0.25	0.55	0.05	0.42	0.75	0.70	0.96	0.15	0.08	0.26	0.67	0.50

	Sarcosine	0.28	0.78	0.58	0.13	0.64	0.27	0.65	0.88	0.69	0.82	0.77	0.22	0.44
	SDMA	0.77	0.58	0.96	0.36	0.53	0.42	0.71	0.57	0.55	0.59	0.98	0.34	0.11
	Serine	0.94	0.08	0.62	0.81	0.39	0.52	0.90	0.08	0.37	0.59	0.09	0.12	0.93
	T4_OH_Pro	0.37	0.55	0.05	0.02	0.70	0.62	0.54	0.78	0.46	0.02	0.95	0.85	0.56
	Taurine	0.51	0.84	0.64	0.24	0.89	0.82	0.38	0.11	0.34	0.35	0.93	0.66	0.65
	Threonine	0.57	0.19	0.69	0.10	0.93	0.47	0.92	0.68	0.32	0.20	0.19	0.39	0.23
	Tryptophan	0.28	0.96	0.76	0.90	0.30	0.71	0.95	0.87	0.02	0.73	0.52	0.43	0.77
	Tyrosine	0.95	0.67	0.49	0.07	0.69	0.98	0.40	0.36	0.76	0.99	0.52	0.70	0.81
	Valine	0.38	0.29	0.73	0.63	0.42	0.55	0.77	0.96	0.76	0.96	0.41	0.11	0.95
Hexose	H1	0.49	0.69	0.14	0.15	0.84	0.06	0.25	0.92	0.71	0.50	0.14	0.44	0.73
Sphingolipids	SM__OH__C14_1	0.88	0.35	0.94	0.68	0.83	0.81	0.93	0.60	0.37	0.38	0.12	0.10	0.83
	SM__OH__C16_1	0.63	0.42	0.48	0.93	0.73	0.64	0.94	0.14	0.57	0.13	0.30	0.32	0.83
	SM__OH__C22_1	0.54	0.82	0.39	0.96	1.00	0.73	0.86	0.16	0.17	0.38	0.07	0.36	0.45
	SM__OH__C22_2	0.42	0.61	0.89	0.75	0.87	0.65	0.96	0.25	0.31	0.30	0.05	0.34	0.84
	SM__OH__C24_1	0.84	0.32	0.96	0.55	0.49	0.61	0.52	0.16	0.07	0.21	0.63	0.25	0.19
	SM_C16:0	0.89	0.78	0.76	0.24	0.92	0.75	0.63	0.77	0.23	0.97	0.29	0.79	0.98
	SM_C16:1	0.66	0.83	0.18	0.02	0.63	0.08	0.51	0.92	0.56	0.07	0.02	0.91	0.77
	SM_C18:0	0.26	0.94	0.87	0.98	0.76	0.41	0.96	0.16	0.50	0.03	0.35	0.27	0.31
	SM_C18:1	0.28	0.78	0.42	0.39	0.88	0.10	0.81	0.16	0.39	0.01	0.20	0.27	0.46
	SM_C20:2	0.74	0.28	0.38	0.17	0.94	0.03	0.47	0.39	0.34	0.52	0.91	0.09	0.54
	SM_C24:0	0.90	0.80	0.54	0.29	0.77	0.48	0.79	0.49	0.11	0.44	0.24	0.70	0.37
	SM_C24:1	0.62	0.51	0.15	0.13	0.76	0.22	0.41	0.62	0.81	0.84	0.74	0.94	0.55
	SM_C26:0	0.44	0.45	0.71	0.90	0.24	0.91	0.96	0.16	0.69	0.38	0.67	0.94	0.29
	SM_C26:1	0.56	0.49	0.11	0.13	0.80	0.53	0.31	0.82	0.70	0.30	0.78	0.76	0.42
Glycerophospholipids	LysoPC_a_C16:0	0.48	0.85	0.61	0.60	0.92	0.84	0.52	0.45	0.83	0.84	0.81	0.03	0.08
	LysoPC_a_C16:1	0.59	0.20	0.17	0.50	0.43	0.38	0.71	0.25	0.88	0.23	0.51	0.02	0.40
	LysoPC_a_C17:0	0.65	0.79	0.68	0.67	0.79	0.32	0.98	0.11	0.27	0.72	0.81	0.74	0.34
	LysoPC_a_C18:0	0.96	0.80	0.69	0.70	0.74	0.80	0.83	0.44	0.67	0.81	0.83	0.55	0.16
	LysoPC_a_C18:1	0.94	0.85	0.29	0.93	0.96	0.88	0.11	0.98	0.52	0.54	0.88	0.03	0.46
	LysoPC_a_C18:2	0.43	0.08	0.57	0.67	0.37	0.46	0.11	0.87	0.94	0.51	0.39	0.98	0.39
	LysoPC_a_C20:3	0.33	0.64	0.86	0.81	0.86	0.92	0.37	0.83	0.55	0.21	0.80	0.10	0.83

LysoPC_a_ C20:4	0.41	0.88	0.19	0.27	0.92	0.13	0.60	0.36	0.63	0.10	0.51	0.28	0.84
LysoPC_a_ C28:1	0.97	0.53	0.85	0.74	0.95	0.33	0.76	0.36	0.07	0.20	0.14	0.66	0.46
PC_ aa _C28:1	0.91	0.66	0.69	0.27	0.61	0.61	0.92	0.72	0.35	0.37	0.08	0.92	0.97
PC_ aa _C30:0	0.58	0.67	0.94	0.57	0.31	0.99	0.47	0.88	0.03	0.28	0.24	0.04	0.81
PC_ aa _C32:0	0.48	0.68	0.65	0.17	0.24	0.35	0.56	1.00	0.10	0.37	0.15	0.08	0.96
PC_ aa _C32:1	0.44	0.21	0.36	0.50	0.18	0.32	0.70	0.12	0.65	0.12	0.24	0.01	0.90
PC_ aa _C32:2	0.88	0.87	0.85	0.51	0.56	0.87	0.42	0.42	0.29	0.93	0.47	0.46	0.96
PC_ aa _C32:3	0.61	0.26	0.38	0.22	0.31	0.93	0.57	0.93	0.30	0.31	0.38	0.97	0.85
PC_ aa _C34:1	0.38	0.27	0.27	0.70	0.16	0.46	0.80	0.45	0.52	0.09	0.17	0.00	0.70
PC_ aa _C34:2	0.81	0.85	0.58	0.42	0.91	0.82	0.88	0.57	0.52	0.47	0.50	0.32	0.34
PC_ aa _C34:3	0.92	0.74	0.30	0.31	0.28	0.94	0.71	0.41	0.38	0.37	0.48	0.18	0.97
PC_ aa _C34:4	0.38	0.67	0.68	0.27	0.22	0.50	0.49	0.60	0.16	0.11	0.16	0.13	0.60
PC_ aa _C36:0	0.59	0.89	0.72	0.86	0.40	0.24	0.43	0.23	0.01	0.83	0.87	0.06	0.75
PC_ aa _C36:1	0.77	0.98	0.33	0.60	0.28	0.92	0.96	0.32	0.39	0.06	0.42	0.01	0.79
PC_ aa _C36:2	0.89	0.83	0.39	0.09	0.60	0.95	0.51	0.49	0.96	0.47	0.58	0.66	0.44
PC_ aa _C36:3	0.25	0.60	0.98	0.27	0.39	0.86	0.90	0.28	0.50	0.28	0.55	0.07	0.62
PC_ aa _C36:4	0.12	0.25	0.36	0.11	0.17	0.06	0.47	0.99	0.64	0.05	0.08	0.03	0.53
PC_ aa _C36:5	0.20	0.88	0.15	0.66	0.32	0.89	0.94	0.71	0.03	0.19	0.53	0.71	0.69
PC_ aa _C36:6	0.64	0.39	0.46	0.99	0.54	0.23	0.68	0.78	0.01	0.34	0.46	0.76	0.64
PC_ aa _C38:0	0.98	0.32	0.74	0.27	0.38	0.56	0.85	0.54	0.02	0.97	0.69	0.07	0.42
PC_ aa _C38:3	0.25	0.58	0.79	0.17	0.21	0.47	0.45	0.44	0.46	0.11	0.49	0.27	0.65
PC_ aa _C38:4	0.16	0.24	0.28	0.02	0.07	0.02	0.25	0.80	0.60	0.05	0.07	0.27	0.34
PC_ aa _C38:5	0.15	0.37	0.19	0.18	0.09	0.33	0.61	0.67	0.04	0.06	0.46	0.23	0.89
PC_ aa _C38:6	0.73	0.70	0.54	0.52	0.56	0.30	0.90	0.30	0.13	0.58	0.26	0.82	0.23
PC_ aa _C40:2	0.94	0.08	0.58	0.77	0.47	0.36	0.87	0.72	0.03	0.24	0.60	0.19	0.86
PC_ aa _C40:3	0.36	0.38	0.87	0.17	0.20	0.72	0.71	0.74	0.00	0.75	0.81	0.61	0.48
PC_ aa _C40:4	0.10	0.18	0.85	0.16	0.10	0.10	0.54	0.41	0.62	0.06	0.07	0.05	0.64
PC_ aa _C40:5	0.28	0.22	0.49	0.19	0.07	0.28	0.49	0.91	0.33	0.05	0.27	0.14	0.74
PC_ aa _C40:6	0.92	0.51	0.38	0.75	0.47	0.36	0.60	0.45	0.26	0.45	0.22	0.41	0.16
PC_ aa _C42:0	0.85	0.17	0.80	0.55	0.84	0.33	0.54	0.20	0.04	0.47	0.12	0.02	0.61
PC_ aa _C42:1	0.80	0.16	0.78	0.43	0.86	0.34	0.67	0.37	0.06	0.15	0.71	0.03	0.84
PC_ aa _C42:2	0.69	0.31	0.99	0.63	0.54	0.45	0.61	0.17	0.02	0.08	0.46	0.02	0.53
PC_ aa _C42:4	0.28	0.64	0.19	0.40	0.21	0.53	0.59	0.99	0.03	0.77	0.53	0.32	0.05
PC_ aa _C42:5	0.19	0.80	0.54	0.16	0.03	0.14	0.57	0.50	0.02	0.70	0.33	0.89	0.82
PC_ aa_ C42:6	0.51	0.18	0.08	0.29	0.58	0.73	0.48	0.46	0.11	0.55	0.46	0.53	0.51
PC_ ae _C30:0	0.64	0.94	0.17	0.92	0.62	0.33	0.71	0.78	0.01	0.83	0.20	0.82	0.17
PC_ ae _C30:2	0.57	0.55	0.17	0.07	0.72	0.82	0.83	0.92	0.33	0.09	0.20	0.18	0.67
PC_ ae _C32:1	0.67	0.73	0.75	0.18	0.85	0.77	0.83	0.35	0.08	0.56	0.27	0.68	0.11
PC_ ae _C32:2	0.70	0.46	0.51	0.06	0.58	0.98	0.37	0.65	0.09	0.13	0.07	0.53	0.09
PC_ ae _C34:0	0.61	0.95	0.88	0.83	0.78	0.32	0.32	0.24	0.01	0.65	0.70	0.60	0.90
PC_ ae _C34:1	0.43	0.60	1.00	0.66	0.53	0.90	0.83	0.57	0.05	0.25	0.19	0.27	0.18

PC_ ae _C34:2	0.13	0.86	0.83	0.28	0.54	0.62	0.41	0.24	0.44	0.18	0.21	0.57	0.07
PC_ ae _C34:3	0.79	0.95	0.90	0.37	0.76	0.57	0.98	0.62	0.17	0.83	0.21	0.63	0.07
PC_ ae _C36:0	0.83	0.92	0.73	0.86	0.43	0.50	0.40	0.70	0.12	0.53	0.68	0.56	0.13
PC_ ae _C36:1	0.39	0.93	0.81	0.78	0.52	0.60	0.59	0.49	0.04	0.31	0.22	0.35	0.81
PC_ ae _C36:2	0.76	0.60	0.45	0.45	0.90	0.35	0.56	0.61	0.27	0.81	0.56	0.31	0.70
PC_ ae _C36:3	0.16	0.69	0.57	0.19	0.76	0.82	0.68	0.22	0.18	0.17	0.41	0.42	0.02
PC_ ae _C36:4	0.03	0.08	0.76	0.03	0.24	0.12	0.62	0.32	0.37	0.01	0.01	0.31	0.07
PC_ ae _C36:5	0.13	0.42	0.24	0.03	0.22	0.28	0.94	0.78	0.17	0.04	0.03	0.48	0.24
PC_ ae _C38:0	0.63	0.46	0.21	0.44	0.27	0.48	0.81	0.70	0.01	0.28	0.57	0.47	0.59
PC_ ae _C38:2	0.93	0.96	0.81	0.19	0.35	0.53	0.52	0.84	0.06	0.95	0.71	0.74	0.80
PC_ ae _C38:3	0.16	0.96	0.35	0.73	0.98	0.52	0.96	0.77	0.25	0.23	0.57	0.78	0.24
PC_ ae _C38:4	0.09	0.51	0.54	0.11	0.38	0.21	0.95	0.92	0.19	0.05	0.02	0.49	0.03
PC_ ae _C38:5	0.19	0.26	0.30	0.00	0.08	0.05	0.78	0.41	0.10	0.03	0.05	0.36	0.04
PC_ ae _C38:6	0.40	0.98	0.35	0.14	0.36	0.94	0.73	0.94	0.04	0.16	0.57	0.61	0.49
PC_ ae _C40:1	0.66	0.60	0.41	0.40	0.30	0.56	0.82	0.57	0.02	0.14	0.55	0.52	0.30
PC_ ae _C40:2	0.65	0.79	0.45	0.85	0.77	0.27	0.62	0.24	0.41	0.43	0.75	0.24	0.57
PC_ ae _C40:3	0.71	0.43	0.09	0.80	0.49	0.16	0.93	0.40	0.21	0.88	0.86	0.35	0.50
PC_ ae _C40:4	0.41	0.97	0.15	0.58	0.71	0.74	0.83	0.47	0.06	0.52	0.10	0.61	0.08
PC_ ae _C40:5	0.42	0.95	0.95	0.50	0.62	0.89	0.73	0.28	0.03	0.28	0.39	0.98	0.28
PC_ ae _C40:6	0.88	0.22	0.84	0.89	0.72	0.19	0.92	0.20	0.01	0.87	0.48	0.05	0.98
PC_ ae _C42:1	0.57	0.89	0.47	0.01	0.02	0.15	0.22	0.93	0.06	0.45	0.20	0.76	0.25
PC_ ae _C42:2	0.68	0.40	0.26	0.69	0.53	0.12	0.66	0.59	0.02	0.83	0.88	0.22	0.44
PC_ ae _C42:3	0.83	0.17	0.14	0.54	0.99	0.04	0.90	0.37	0.03	0.31	0.23	0.12	0.99
PC_ ae _C42:4	0.38	0.76	0.04	0.94	0.66	0.57	0.85	0.36	0.10	0.84	0.88	0.54	0.35
PC_ ae _C42:5	0.58	1.00	0.67	0.33	0.68	0.91	0.69	0.65	0.13	0.50	0.78	0.61	0.34
PC_ ae _C44:3	0.19	0.24	0.26	0.27	0.65	0.25	0.42	0.34	0.04	0.30	0.66	0.17	0.40
PC_ ae _C44:4	0.95	0.53	0.15	0.88	0.40	0.25	0.77	0.94	0.34	0.82	0.48	0.42	0.63
PC_ ae _C44:5	0.43	0.71	0.44	0.18	0.76	0.69	0.71	0.98	0.41	0.98	0.71	0.54	0.49
PC_ ae _C44:6	0.87	0.44	0.84	0.14	0.76	0.77	0.98	0.57	0.07	0.61	0.55	0.03	0.37

C0 Carnitine; C12 Decanoylcarnitine; C12:1 Decenoylcarnitine; C14 Tetradecanoylcarnitine; C14:1 Tetradecenoylcarnitine; C16 Hexadecanoylcarnitine; C18 Octadecanoylcarnitine; C18:1 Octadecenoylcarnitine; C18:2 Octadecadienylcarnitine; C2 Acetylcarnitine; C3 Propionylcarnitine; C4 Butyrylcarnitine; C5 Valerylcarnitine

ADMA Asymmetric dimethylarginine

SDMA Symmetric dimethylarginine

T4_OH_Pro Trans-4-Hydroxyproline

LysoPC_a Lyso-phosphatidylcholines

PC_aa Phosphatidylcholines di-alkyl

PC_ae Phosphatidylcholines alkyl-acyl

Table S3. Metabolites with significantly different mean concentrations by age categories in the PRÄVENT cohort (N=108).

Metabolite class	Metabolite	p-value*
Acylcarnitines	C3	0.004500
	C12:1	0.003000
	C14	0.009375
Amino Acids and Biogenic Amines	Alanine	0.009750
	Citrulline	0.000750
	Creatinine	0.002250
	Glutamate	0.010125
	Kynurenine	0.000375
	Phenylalanine	0.006375
	Proline	0.007500
	Sarcosine	0.008250
	SDMA	0.003375
	Tyrosine	0.001500
Sphingolipids	SM__OH__C14:1	0.004875
	SM__OH__C16:1	0.005625
	SM__OH__C22:1	0.006000
	SM__OH__C22:2	0.010875
	SM__OH__C24:1	0.001125
	SM_C16:0	0.007875
	SM_C16:1	0.005250
	SM_C26:1	0.001875
Glycerophospholipids	PC_aa_C28:1	0.004125
	PC_ae_C30:2	0.003750
	PC_aa_C36:5	0.006750
	PC_aa_C36:6	0.010500
	PC_aa_C38:5	0.007125
	PC_ae_C40:2	0.009000
	PC_aa_C40:3	0.008625
	PC_aa_C40:6	0.002625

*FDR adjusted

C3 Propionylcarnitine; C12:1 Decenoylcarnitine; C14 Tetradecanoylcarnitine

SDMA Symmetric dimethylarginine

PC_aa Phosphatidylcholines di-alkyl

Table S4. Specific metabolites with significant differences in mean metabolite concentrations by sex categories in the PRÄVENT cohort (N=108).

Metabolite class	Metabolite	p-value*
Acylcarnitines	C4	0.0071250
	C16	0.0033750
	C18	0.0063750
Amino Acids and Biogenic Amines	Creatinine	0.0003750
	Glutamine	0.0011250
	Sarcosine	0.0056250
	SDMA	0.0030000
	Serine	0.0041250
	Valine	0.0045000
Sphingolipids	SM__OH__C22:2	0.0018750
	SM_C16:1	0.0026250
	SM_C18:1	0.0022500
Glycerophospholipids	PC_ae_C32:1	0.0052500
	PC_ae_C32:2	0.0007500
	PC_ae_C34:3	0.0060000
	PC_ae_C40:3	0.0015000
	PC_ae_C42:3	0.0048750
	PC_ae_C42:4	0.0037500
	PC_ae_C42:5	0.0067500

*FDR adjusted

C4 Butyrylcarnitine; C16 Hexadecanoylcarnitine; C18 Octadecanoylcarnitine

SDMA Symmetric dimethylarginine

PC_ae Phosphatidylcholines alkyl-acyl
